# Single nucleotide polymorphisms in *TNFAIP3* were associated with the risks of rheumatoid arthritis in northern Chinese Han population

**DOI:** 10.1186/1471-2350-15-56

**Published:** 2014-05-15

**Authors:** Xingang Zhang, Wei Li, Xinpeng Zhang, Liang Zhao, Xiaoli Zhang, Li Jiang, Yun Guo, Jin Zhang, Zaifu Liang, Xiaofei Wang

**Affiliations:** 1 Department of Rheumatology, Shengjing Hospital of China Medical University, Shenyang, Liaoning Province, China; 2 Editorial Department of Chinese Pediatric Emergency Medicine, Shenyang, Liaoning Province, China; 3 Department of Neurosurgery, The Fourth People’s Hospital of Shenyang City, Shenyang, Liaoning Province, China; 4 Peking University Health Science Center, Peking, China; 5 Cord Blood Stem Cell Bank of Liaoning Province, Shenyang, Liaoning Province, China; 6 The Center of Laboratory Technology and Experimental Medicine of China Medical University, Shenyang, Liaoning Province, China

**Keywords:** *TNFAIP3* gene, Single nucleotide polymorphism, Rheumatoid arthritis, Risk, Genetic susceptibility

## Abstract

**Background:**

Rheumatoid arthritis (RA) is a systemic autoimmune disease characterized by chronic destructive inflammation in synovial joints. It is well known that genetic and environmental risk factors and their interaction contribute to RA pathogenesis. This study aimed to investigate the association between the critical polymorphisms in the *tumor necrosis factor α (TNFα)-induced protein 3(TNFAIP3)* gene and the risk of RA in a large northern Chinese Han population.

**Methods:**

A case–control study of 1280 RA patients and 1280 matched healthy controls was conducted.

**Results:**

This study showed that carriers of the rs2230926 TG genotype or rs10499194 CT genotype had an increased risk for RA compared with those carrying the wild genotype (rs2230926: OR = 1.48, 95% CI = 1.17-1.86, *p* = 0.001; rs10499194: OR = 2.00, 95% CI = 1.46-2.74, *p* < 0.001). The combined rs2230926TG/GG or rs10499194 CT/TT were associated with an increased risk of RA (ORs were 1.50 and 2.01, 95% CIs were 1.19-1.88 and 1.47-2.74, respectively, both *p* < 0.001). There was not significant association between rs13207033 polymorphism and RA risk. Subset analysis stratified to gender showed that the increased risks were significant among the genotypes TG, TG/GG of rs2230926 and CT, CT/TT of rs10499194 and the corresponding ORs were 1.42 (95%  CI = 1.10-1.83, *p* = 0.006), 1.44(95% CI = 1.12-1.85, *p* = 0.004), 1.52(95% CI = 1.05-2.20, *p* = 0.026) and 1.52(95% CI = 1.06-2.19, *p* = 0.023) in the female population. Stratified analyses by age found that rs2230926(TG, TG/GG) and rs10499194(CT, CT/TT) polymorphisms were associated with RA risks in population ≤53 years old and among >53 years old only rs10499194(CT, TT, CT/TT) polymorphism had significant results. The interaction analysis suggested that individuals with both risk genotypes of the two SNPs have a higher elevated risk of RA than those with only one of them (ORs were 3.44 compared to 1.74 and 1.35). The haplotype results showed that individuals with the rs2230926G-rs13207033G-rs10499194C haplotype were associated with increased risks of RA (OR = 1.37, 95% CI = 1.08-1.74, *p* = 0.010).

**Conclusions:**

Rs10499194 and rs2230926 polymorphisms in the *TNFAIP3* gene region may be susceptibility factors for rheumatoid arthritis in the northern Chinese Han population.

## Background

Rheumatoid arthritis (RA) is a systemic autoimmune disease. It is characterized by chronic destructive inflammation in synovial joints. It is well known that genetic and environmental risk factors and their interaction contribute to RA pathogenesis [1]. So far the etiology is still not understood, but the genetic factors significantly contribute to RA with heritability estimated more than half(53-60%) [2]. The key role of nuclear factor-κB (NF-κB)-dependent gene expression in the development of autoimmune diseases including RA is generally accepted. TNFAIP3(A20), as a deubiquitinating protein, can deregulate NF-κB-dependent gene expression and inhibit NF-κB function by deubiquitinating specific NF-κB signaling molecules [3,4]. Defects in A20 expression are associated with development of many human autoimmune diseases [5]. The development of severe inflammation of the joints are found in A20-deficient mice [6]. A20 deficiency in myeloid cells spontaneously develops a severe destructive polyarthritis with many features of RA in mice [7]. Loss of A20 in B cells causes inflammation and leads to autoimmune pathology in old mice [8]. In addition, Kool et al. found that A20-mediated control of dendritic cells (DC) activation as a crucial checkpoint in the development of systemic autoimmunity [9]. DCs need A20 to preserve immune quiescence and prevents colitis and spondyloarthritis in mice [10].

More recently, studies shown that several single nucleotide polymorphisms (SNPs) in human TNFAIP3 loci involve in increased susceptibility to RA [5]. The associated SNPs (rs6920220 and rs10499194) are located near oligodendrocyte transcription factor 3(OLIG3) and TNFAIP3 genes [6]. Studies in Caucasian populations identified that SNPs in the candidate loci, such as rs6920220, rs13207033, rs582757 and et al., are associated with RA [4,11-13]. Rs10499194 was initially identified in North American ACPA + patients [12]. An association between F127C and A125V human polymorphism in TNFAIP3 and autoimmunity in African Americans was reported by Lodolce JP. et al. [14]. The majority of RA risk alleles previously validated for RA patients of European ancestry showed similar ORs in the population of African Americans with RA [15]. Studies which tested the 6q23 region in Tunisian population revealed a trend of an association of rs6920220-A allele with RA and genotypes containing this allele were in a higher proportion in RA patients than in matched controls [16]. Lately the association of TNFAIP3 SNPs with RA was replicated in Japanese and Korean populations [17,18].

To identify the role of the polymorphisms in the TNFAIP3 gene on the risk of RA in Asian population, in the present study, we describe a case–control study of 1280 RA cases and 1280 controls from northern Chinese han population; stratified analyses were carried on by gender and age; we also explored the interaction between genetic polymorphisms in the development of RA.

## Methods

### Study subjects and data collection

The present case–control study, conducted between July 2010 and December 2013, included 1280 diagnosed RA patients and 1280 healthy controls. All RA cases were selected following the diagnostic features of RA established by the American College of Rheumatology criteria [19]. At the same time, unrelated healthy control subjects without any indication of RA were selected. Controls were all frequency matched to cases on age (±3 years) and gender. The human investigations were approved by the Ethics Committee of Shengjing Hospital of China Medical University, and informed consents were obtained from each participant. All patients were unrelated ethnic Han Chinese. Each participant donated 2 ml venous blood and was interviewed to collect the demographic data. Three SNPs of the *TNFAIP3* gene were analyzed in the present study: rs10499194, rs2230926 and rs13207033.

### DNA isolation and genotyping

Genomic DNA samples were isolated using the phenol–chloroform method. SNPs were analyzed by TaqMan assays. For the selected SNPs, designed TaqMan SNP genotyping assays were used. Primers and probes were synthesized by Shanghai Generay Biotechnology Co, Ltd. (P.R. China). The sequences of probes and primers are listed as follows: rs2230926, forward primer: GAT TTG AGA GAC TCC AGT TGC CA, reverse primer: GCG TTC AGG ACA CAG ACT TGG, probe 1: FAM-AGC GTG CTG AAC AGC GCC TTC-TAMRA, probe 2: HEX-AGC GTG CTG CAC AGC GCC T-TAMRA; rs13207033, forward primer: GCA CAA TGA AAG AGA GAG AAG TAG AAT AAT, reverse primer: ATC AAC ATT TGT CTA TTT TAT GCT CCA, probe 1: FAM-TAC AGA TTT CAC TTT CCC T-MGB, probe 2: HEX-CTA CAG ATT TTA CTT TCC-MGB; rs10499194, forward primer: GCT ATC AGT TTC ATT ACC TAA GAA ATA GAG AC, reverse primer: CAA CGG ATA AGC AAT CAG ACC A, probe 1: FAM-AAT GTG TTC AAC CCT TT-MGB, probe 2: HEX-CAA ATG TGT TTA ACC CTT T-MGB. Fluorescence was detected using an ABI Prism 7500 Sequence Detection System (Applied Biosystems). A 10% masked random sample of subjects was tested twice by different persons for quality control, and the results were found to be in agreement for all of the duplicate sets.

### Statistical analysis

Pearson’s chi-square test was used to compare the distribution of the demographic variables and SNP genotypes between cases and controls. Hardy-Weinberg equilibrium (HWE) of the genotypes was tested by performing a goodness-of-fit *χ*^2^ test. Unconditional logistic regression analysis was performed to calculate the odds ratios (OR) with 95% confidence intervals (CI) for estimating the association between certain genotypes and RA. The interactions were evaluated by cross-over analysis and logistic regression models. All statistical analyses were performed with SPSS (v16.0), if not otherwise specified. All of the tests were two-sided, and statistical significance was defined as *p* < 0.05. On the basis of the observed frequencies of the three SNPs, we used the SHEsis analysis platform to calculate linkage disequilibrium indices (D’ and r^2^) and infer haplotype frequencies [20,21].

## Results

In total, 1280 RA patients and 1280 healthy controls were included in the present study. There were 1088 females and 192 males in the case group, and 1051 females and 229 males in the control group. The mean ages of the cases and controls (mean ± S.D.) were almost identical (53.99 ± 13.61 and 53.52 ± 8.63 years, respectively). The distributions of gender or age between the case and control groups were identical.

The frequencies of the rs2230926 G allele, rs13207033 A allele and rs10499194 T allele were 0.059, 0.097, 0.030, respectively, in the controls and 0.084, 0.097, 0.053, respectively, in the cases. All allele distributions were consistent with HWE (*p* values were 0.819, 0.203, 0.218 in controls and 0.687, 0.200, 0.638 in cases for the SNPs, respectively).

Table 1 present the distribution of genotypes for polymorphisms in the *TNFAIP3* gene in cases and controls. Among these SNPs, rs2230926 and rs10499194 were associated with the risks of RA, however the rs13207033 polymorphism was not associated with RA risk in this study. The heterozygous carriers of the rs2230926 TG genotype had a 1.48-fold risk of RA compared with the homozygous wild genotype TT (95% CI = 1.17-1.86, *p* = 0.001). Individuals carrying the rs10499194 CT heterozygous genotype had a 2.00-fold increased risk of RA compared with the homozygous wild genotype CC (95% CI = 1.46-2.74, *p* < 0.001). Analyses combining the heterozygous variant genotype with the homozygous variant genotype showed that the combined rs2230926 TG/GG and rs10499194 CT/TT were associated with increased risks of RA (ORs were 1.50 and 2.01, 95% CIs were 1.19-1.88 and 1.47-2.74, respectively, both *p* < 0.001). The overall ORs with its 95% CIs didn’t show statistically association between rs13207033 polymorphism and RA risk (GA vs. GG: OR = 1.08, 95% CI = 0.88-1.33, *p* = 0.448; AA vs. GG: OR = 0.50, 95% CI = 0.22-1.18, *p* = 0.115; GA + AA vs. GG: OR = 1.04, 95% CI = 0.85–1.27, *p* = 0.683).

**Table 1:** **Association between polymorphisms in *TNFAIP3 *** **gene and rheumatoid arthritis in northern Chinese han subjects**

**Genotype**	**Cases (%)**	**Controls (%)**	**OR (95% CI)**	***p *** **value**
rs2230926				
TT	1072(83.8)	1133(88.5)	1.00	—
TG	200(15.6)	143(11.2)	1.48(1.17-1.86)	0.001
GG	8(0.6)	4(0.3)	2.11(0.64-7.04)	0.223
TG/GG	208(16.2)	147(11.5)	1.50(1.19-1.88)	<0.001
rs13207033				
GG	1040(81.3)	1048(81.9)	1.00	—
GA	232(18.1)	216(16.9)	1.08(0.88-1.33)	0.448
AA	8(0.6)	16(1.2)	0.50(0.22-1.18)	0.115
GA/AA	240(18.7)	232(18.1)	1.04(0.85-1.27)	0.683
rs10499194				
CC	1156(90.3)	1215(94.9)	1.00	—
CT	120(9.4)	63(4.9)	2.00(1.46-2.74)	<0.001
TT	4(0.3)	2(0.2)	2.10(0.38-11.50)	0.392
CT/TT	124(9.7)	65(5.1)	2.01(1.47-2.74)	<0.001

Data were calculated by unconditional logistic regression.

Considering that RA occurred more often in females, we conducted a subset analysis in Chinese female population (Table 2). The results showed that the increased risks were significant among the rs2230926 variant genotypes (TG) and rs10499194 variant genotypes (CT) and the corresponding ORs were 1.42 (95% CI = 1.10-1.83, *p* = 0.006) and 1.52 (95% CI = 1.05-2.20, *p* = 0.026), respectively. The subjects with TG or GG genotype of rs2230926 and CT or TT genotype of rs10499194 were in higher risks to develop RA (ORs were 1.44 and 1.52, 95% CIs were 1.12-1.85 and 1.06-2.19, *p* = 0.004 and 0.023, respectively). Significant associations were not seen in the rs13207033 polymorphism (GA vs. GG: OR = 1.07, 95% CI = 0.85-1.35, *p* = 0.583; AA vs. GG: OR = 0.77, 95% CI = 0.30-1.96, *p* = 0.587; GA + AA vs. GG: OR = 1.05, 95% CI = 0.84–1.32, *p* = 0.674).

**Table 2:** **Polymorphisms in *TNFAIP3 *** **gene and risks of rheumatoid arthritis in females**

**Genotype**	**Cases (%)**	**Controls (%)**	**OR [95% CI]**	***p *** **value**
rs2230926				
TT	912(83.8)	918(88.2)	1.00	
TG	168(15.5)	119(11.4)	1.42[1.10-1.83]	0.006
GG	8(0.7)	4(0.4)	2.01[0.60-6.71]	0.255
TG/GG	176(16.2)	123(11.8)	1.44[1.12-1.85]	0.004
rs13207033				
GG	904(83.1)	872(83.8)	1.00	
GA	176(16.2)	159(15.2)	1.07[0.85-1.35]	0.583
AA	8(0.7)	10(1.0)	0.77[0.30-1.96]	0.587
GA/AA	184(16.9)	169(16.2)	1.05[0.84-1.32]	0.674
rs10499194				
CC	1009(92.7)	990(94.2)	1.00	
CT	76(7.0)	49(4.6)	1.52[1.05-2.20]	0.026
TT	3(0.3)	2(0.2)	1.47[0.25-8.83]	0.672
CT/TT	79(7.3)	51(4.8)	1.52[1.06-2.19]	0.023

Furthermore, stratified analyses were adopted by age and the population was divided into two groups for ≤53 years old and >53 years old (Table 3). Age 53 was selected because it is the mean age of the present population and sample size is reasonable in stratified groups. For rs2230926 polymorphism, the significant results were found in ≤53 years old group (TG vs. TT: OR = 1.73, 95% CI = 1.26-2.39; TG + GG vs. TT: OR = 1.80, 95% CI = 1.31-2.47), but not in > 53 years old (TG vs. TT: OR = 1.30, 95% CI = 0.93-1.80; TG + GG vs. TT: OR = 1.28, 95% CI = 0.92-1.76). In terms of rs10499194 polymorphism, the associations were significant in both ≤53 years old and >53 years old groups. Individuals carrying the CT genotype were more likely to develop RA among people ≤53 years old (OR = 1.89, 95% CI = 1.24-2.90) and >53 years old(OR = 2.25, 95% CI = 1.39-3.63). Individuals with the TT genotype was more likely to develop RA among people >53 years old(OR = 2.81, 95% CI = 0.29-27.07). The significant results were also seen in CT/TT with ORs 1.88 and 2.27, 95% CIs 1.24-2.86 and 1.42-3.63, for the subjects ≤53 years old and >53 years old, respectively. For rs13207033 polymorphism, the results in two stratified groups were all not significant.

**Table 3:** Polymorphisms in TNFAIP3 gene and risks of rheumatoid arthritis stratified by age

**Genotype**	**≤53**			**>53**		
	**Cases (%)**	**Controls (%)**	**OR [95% CI]**	**Cases (%)**	**Controls (%)**	**OR [95% CI]**
rs2230926						
TT	452(80.7)	564(88.3)	1.00	620(86.1)	569(88.8)	1.00
TG	104(18.6)	75(11.7)	1.73[1.26-2.39]	96(13.3)	68(10.6)	1.30[0.93-1.80]
GG	4(0.7)	0(0.0)	—	4(0.6)	4(0.6)	0.92[0.23-3.69]
TG/GG	108(19.3)	75(11.7)	1.80[1.31-2.47]	100(13.9)	72(12.2)	1.28[0.92-1.76]
rs13207033						
GG	472(84.3)	528(82.6)	1.00	568(78.9)	520(81.1)	1.00
GA	84(15.0)	107(16.7)	0.88[0.64-1.20]	148(20.6)	109(17.0)	1.24[0.95-1.64]
AA	4(0.7)	4(0.6)	1.12[0.28-4.50]	4(0.6)	12(1.9)	0.31[0.10-0.95]
GA/AA	88(15.7)	111(17.4)	0.89[0.65-1.20]	152(21.1)	121(18.9)	1.15[0.88-1.50]
rs10499194						
CC	499(89.1)	600(93.9)	1.00	657(91.3)	615(95.9)	1.00
CT	60(10.7)	38(5.9)	1.89[1.24-2.90]	60(8.3)	25(3.9)	2.25[1.39-3.63]
TT	1(0.2)	1(0.2)	1.20[0.08-19.27]	3(0.4)	1(0.2)	2.81[0.29-27.07]
CT/TT	61(10.9)	39(6.1)	1.88[1.24-2.86]	63(8.7)	26(4.1)	2.27[1.42-3.63]

Our results revealed a significant association between the risk of RA and both rs2230926 and rs10499194, when comparing allele frequency in patients and control subjects (ORs were 1.47 and 1.96, 95% CIs were 1.19-1.82 and 1.45-2.65, respectively, both *p* < 0.001) (Table 4).

**Table 4:** Allele frequencies of polymorphisms in TNFAIP3 gene and risk of rheumatoid arthritis in northern Chinese han subjects

**dbSNP number, major/minor allele**	**Allele frequency**		**OR (95% CI)**	***p *** **value**
	**Cases**	**Controls**		
rs2230926, T/G	0.916/0.084	0.941/0.059	1.47(1.19-1.82)	<0.001
rs13207033, G/A	0.903/0.097	0.903/0.097	1.00(0.83-1.20)	0.999
rs10499194, C/T	0.947/0.053	0.970/0.030	1.96(1.45-2.65)	<0.001

We evaluated the interaction of rs2230926 and rs10499194 on the risk of RA using cross-over analysis and logistic regression model (Table 5). The results suggested that individuals with both risk genotypes of the two SNPs have a higher elevated risk of RA than those with only one of them (ORs were 3.44 compared to 1.74 and 1.35).

**Table 5:** Interaction of rs2230926 and rs10499194 on risk of rheumatoid arthritis in northern Chinese han subjects

**rs2230926**	**rs10499194 CC CT**			
	**Cases/Controls**	OR (95% CI)*	**Cases/Controls**	**OR (95% CI)**
TT	989/1080	1.00	83/52	1.74(1.22-2.49)
TG/GG	167/135	1.35(1.06-1.72)	41/13	3.44(1.84-6.47)

*OR and 95% CI were calculated by logistic regression, with the rs2230926 wild genotype (TT) as the reference group.

Table 6 showed the haplotype analysis results. The three SNPs were in linkage disequilibrium in the present population. Three of eight possible haplotypes had a frequency of >0.03 among both cases and controls and were included in the haplotype analysis. Three possible haplotypes represented 94.3% of the chromosomes for the cases and 96.8% for the controls. According to our priori hypothesis and the SNP-based analyses, we considered the individuals with the rs2230926T-rs13207033G-rs10499194C haplotype to be the reference group for OR estimations. The G-G-C haplotype was associated with increased risks of RA (OR = 1.37, 95% CI = 1.08-1.74, *p* = 0.010).

**Table 6:** Association of haplotypes and risk of rheumatoid arthritis

**Haplotype**	**Cases (%)**	**Controls (%)**	**OR (95% CI)**	***p *** **value**
T-G-C	2108(82.3)	2178(85.1)	1.00	
T-A-C	142(5.5)	174(6.8)	0.84(0.67-1.06)	0.145
G-G-C	167(6.5)	126(4.9)	1.37(1.08-1.74)	0.010

## Discussion

The 6q23 region has recently been associated with disease susceptibility in RA. This region contains no known transcripts. The clearest candidate gene among the flanking of this locus with known function is *TNFAIP3* gene. *TNFAIP3* encodes protein A20, a TNFα-induced negative regulator of NF-κB activity, resulting in increased inflammation. This observation makes *TNFAIP3*/A20 and the 6q23 region interesting candidates which may modulate inflammation also in RA. Therefore, we studied the association of SNPs in *TNFAIP3* gene and RA risks in a large northern Chinese han population. Our results suggest that polymorphisms in the *TNFAIP3* gene were associated with RA susceptibility. Therefore, it seems possible that this region contains some genetic factors involved in RA.

To date, the majority of GWAS and subsequent meta-analyses of GWAS data in RA have focused on individuals of European and East Asian ancestry. It has become clear from these and other large-scale genetic studies of complex diseases such as RA that genetic risk loci can differ between these different ethnic groups [22-24]. In the present study, we sought to test the hypothesis that RA risk SNPs validated in populations of other country would also be associated with RA in a large northern Chinese han population.

The GWAS conducted in the WTCCC study and a replication study using the WTCCC data demonstrated associated SNPs for RA at 6q23 [12,25]. Studies in the Caucasian population have consistently shown that SNPs at the *TNFAIP3* locus might increase the susceptibility to RA [26,27]. Rs2230926 and rs10499194 polymorphisms have been reported to be closely associated with risk of RA in Japanese subjects [18]. In contrast, Han et al. have not found the significant association between rs10499194 polymorphism and the RA risk in Korean population [17]. Furthermore, it reported no association between rs10499194 and RA in African-Americans [15]. Our study found the significant association between RA and the TNFAIP3 gene variants. In the present study, rs2230926, located in exon 3 of *TNFAIP3,* was significantly associated with a predisposition to RA in northern Chinese han subjects. SNP rs2230926 (T/G) is a nonsynonymous variant that results in a phenylalanine-to-cysteine change at residue 127 of the A20 protein. It is reported that A20 127Cys protein (encoded by the risk allele of G) was less effective at inhibiting TNF-induced NF-κB activity than the Phe127 protein (encoded by the T allele) [28], which suggests that reduced negative regulatory activity of the A20 protein may allow excessive immune activity, leading to enhanced autoreactivity. SNP rs10499194, one of the landmark polymorphisms identified in Caucasian patients with RA [12,26], was suggested to be a susceptibility factor of RA in our population. But its significant association with RA could not be replicated in another Caucasian population [29]. Rs10499194 was also showed to be associated with RA susceptibility in Japanese population [18]. Because rs10499194 is also associated with RA susceptibility in our population, rs10499194 might be a landmark for disease causal variants in northern Chinese han patients with RA. However, for another SNP in the 6q23 intergenic region, rs13207033, we found no association with RA risk. This is in agreement with another study [13]. On the other hand, previous report supported the significant association of the rs13207033 SNP with RA [12,25]. The reason for these different conclusions is not clear now. There are at least 3 explanations for these differences. First, it might be attributable to weak correlations between SNPs and actual risk of RA. It is known from the International HapMap Project that the linkage disequilibrium structure between European and African-American populations may be different for common SNPs in any given locus. Such an effect may be particularly striking if the causal allele is rare in the population (e.g., frequency 5%). Second, these differences may be explained by genetic heterogeneity; a risk allele in European patients with RA may have different effect in Asians. Such population-specific differences may also reflect gene–gene or gene–environment interactions. Third, the inconsistency may simply be attributable to chance. Thus, further analysis in other sample populations will be required to confirm the association between SNPs in *TNFAIP3* gene and RA risks. The exact effects and mechanisms of these polymorphisms on RA need further studies to elucidate.

In summary, our study sheds light on the relationship between polymorphisms in *TNFAIP3* gene and the susceptibility to RA in northern Chinese han population. Our results show that the SNPs rs2230926 and rs10499194 may be strong causal variant candidates of RA in this region. While the functional interpretation remains elusive, a search for additional causal variants in *TNFAIP3* is required. Moreover, combination of genetic factors together with environmental exposures should also be considered.

## Conclusions

Rs10499194 and rs2230926 polymorphisms in the *TNFAIP3* gene region may be susceptibility factors for rheumatoid arthritis in the northern Chinese Han population.

## Competing interests

The authors declare that they have no competing interests.

## Authors’ contributions

XZ conceived and designed the article, collected the cases and acquisited the data, carried out the molecular epidemiological studies, extracted and detected DNA, recruited the controls and acquisited the data, sorted and analysed the data. WL carried out the molecular epidemiological studies, extracted and detected DNA, recruited the controls and acquisited the data, sorted and analysed the data. XZ carried out the molecular epidemiological studies, extracted and detected DNA, recruited the controls and acquisited the data, sorted and analysed the data. LZ collected the cases and acquisited the data, recruited the controls and acquisited the data. XZ collected the cases and acquisited the data. LJ collected the cases and acquisited the data. YG collected the cases and acquisited the data. JZ carried out the molecular epidemiological studies, extracted and detected DNA. ZL conceived and designed the article. XW conceived and designed the article. All authors read and approved the final manuscript.

## Pre-publication history

The pre-publication history for this paper can be accessed here:

http://www.biomedcentral.com/1471-2350/15/56/prepub
